# Supplementary material for: An essential role for actinotrichia in zebrafish fin patterning and courtship behavior
Source: Development. 2026 Apr 15;153(16):dev204990. doi: 10.1242/dev.204990 (PMC13120675; doi:10.1242/dev.204990)
Supplement: Supplementary information [file develop-153-204990-s1.pdf]

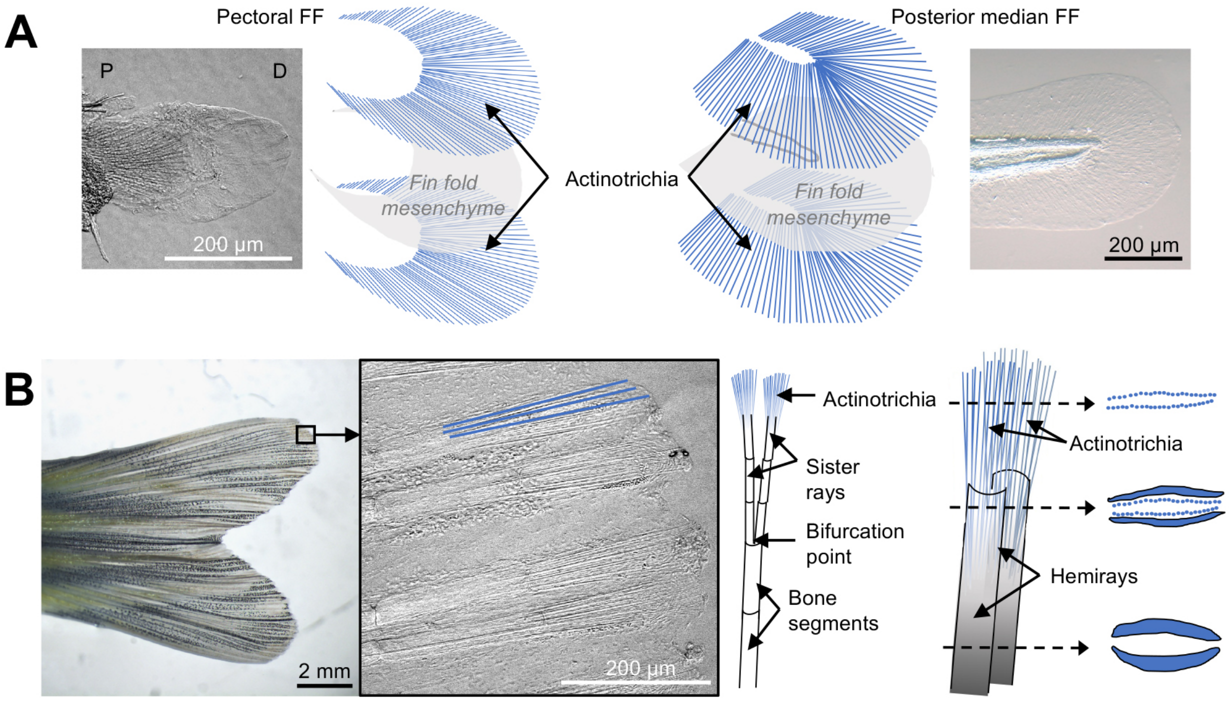

Fig. S1. Schematic representation of actinotrichia within the fin folds and in the adult rays.

(A) Actinotrichia within the pectoral fin fold (FF) (left) and posterior median FF (right), represented at 7 dpf. Actinotrichia (blue lines) are found in two parallel arrays in the FF mesenchyme. P = Proximal; D = Distal. (B) Actinotrichia are found at the tips of the rays in adult fins, represented in a caudal fin (left). Principal rays are made of two concave segmented hemirays, and bifurcate distally into two sister rays. Actinotrichia are found at the distal tips, in two bundles, overlapping with the distalmost bone segments. Rightmost schematic shows cross-sectional view of distal tip of the ray, with positions indicated with dashed black arrows.

Development • Supplementary information

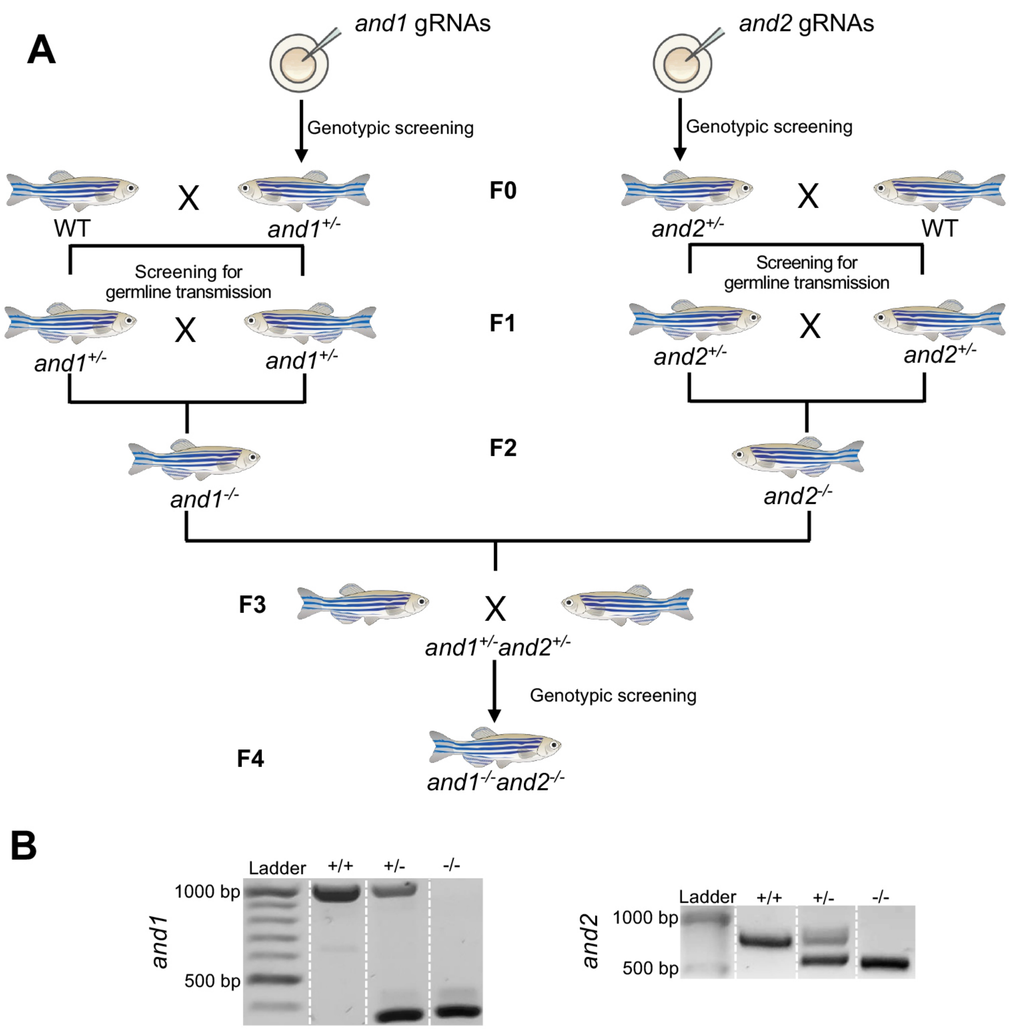

Fig. S2. Generation of *and1$^{-/-}$and2$^{-/-}$* double mutants by CRISPR-Cas9.

(A) Schematic representation of the generation of the *and1$^{-/-}$and2$^{-/-}$* double mutant line. Zebrafish were raised to three months between generations and selectively bred to generate single heterozygotes (F2). Single heterozygotes for *and1* and *and2* were crossed to obtain double heterozygotes (F3), which were inbred to produce double homozygotes (F4). Zebrafish icon by DBCLS https://togotv.dbcls.jp/en/pics.html is licensed under CC-BY4.0 Unported https:// creativecommons.org/licenses/by/4.0/. embryo_gene-injection icon by DBCLS https://togotv.dbcls. jp/en/pics.html is licensed under CC-BY 4.0 Unported https://creativecommonsorg/licenses/ by/4.0/

(B) PCR products of *and1* (left) and *and2* (right) amplification for genotypic screening. A GeneRuler 1kb DNA ladder was used. The *and1* WT fragment is 1055 bp, the *and1* deletion fragment is 386 bp, the *and2* WT fragment is 848 bp, and the *and2* deletion fragment is 579 bp. Dashed lines indicate where lanes were cropped.

Development • Supplementary information

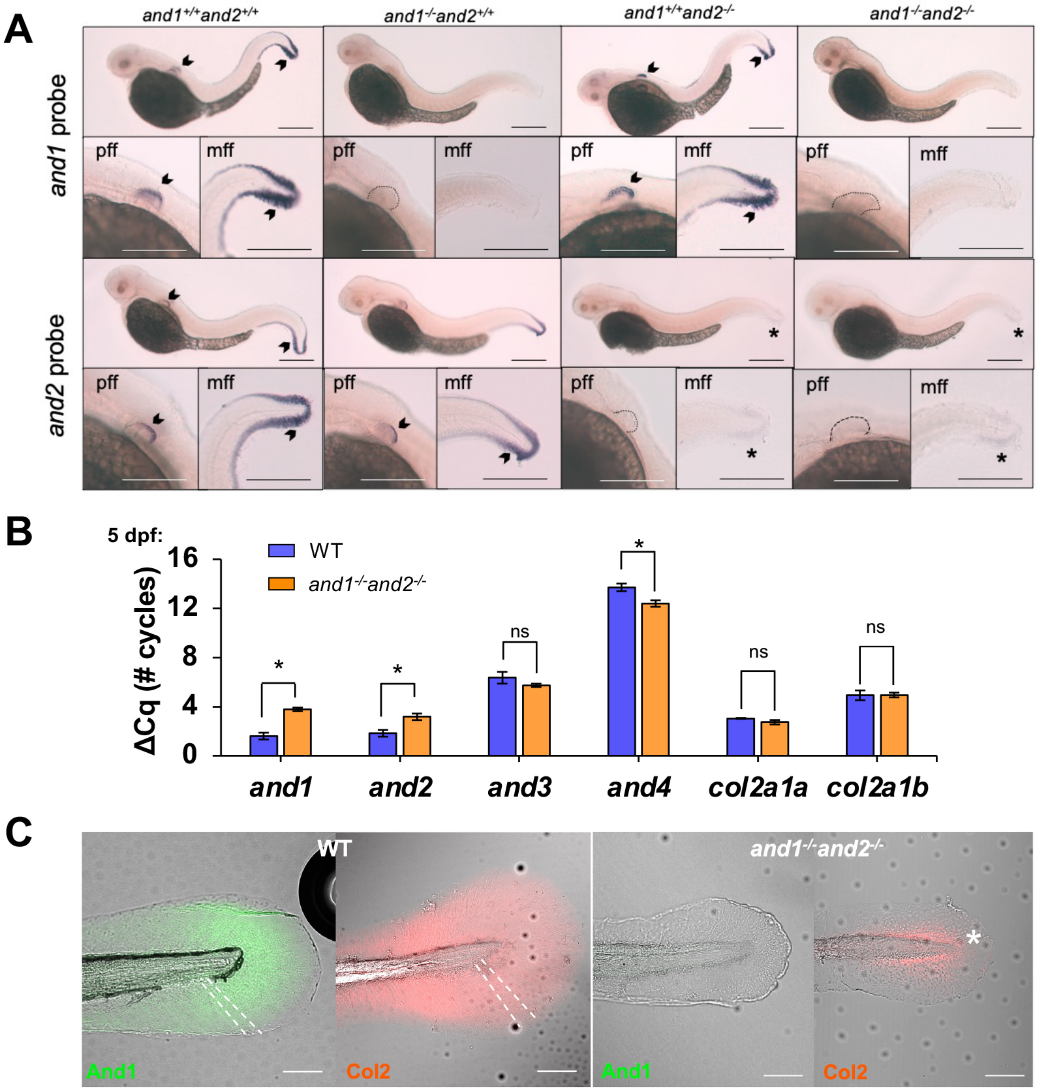

Fig. S3. Embryonic and larval *and1* and *and2* expression analysis and actinotrichia visualization.

(A) Whole mount *in situ* hybridization of *and1* and *and2* antisense riboprobes of WT (n_{and1} = 10; 32n_{and2} = 10), $and1^{-/-}and2^{+/+}$ (n_{and1} = 6; n_{and2} = 5), $and1^{-/-}and2^{+/+}$ (n_{and1} = 5; n_{and2} = 5), and double homozygous (n_{and1} = 4; n_{and2} = 5) embryos at 2 dpf. Embryos were stained for 60 minutes. Black arrowheads indicate purple staining in mff and pff. Pectoral fin folds lacking expression are emphasized with black dotted outline. Black asterisks indicate trace expression in median fin folds. Scale bars: 250 µm.

(B) Mean ΔCq of *and1*, *and2*, *and3*, *and4*, *col2a1a*, and *col2a1b* expression in WT sibling and double mutant tails at 5 dpf (n = 20 tails per biological replicate). Error bars: Standard error of mean ΔCq. Statistics: unpaired two-tailed t-test, $p < 0.05$ (*).

(C) Merged fluorescent (shown alone in Figure 2B) and TD channel images of And1- and Col2-immunostained WT sibling (n = 6 larvae) and double mutant (n = 6 larvae) posterior median FFs at 5 dpf. Dashed lines represent actinotrichia in the WT siblings. Actinotrichia are absent in the double mutant median FF, shown by lack of And1 signal, and disorganized and aggregating Col2. Scale bars: 100 μm.

Development • Supplementary information

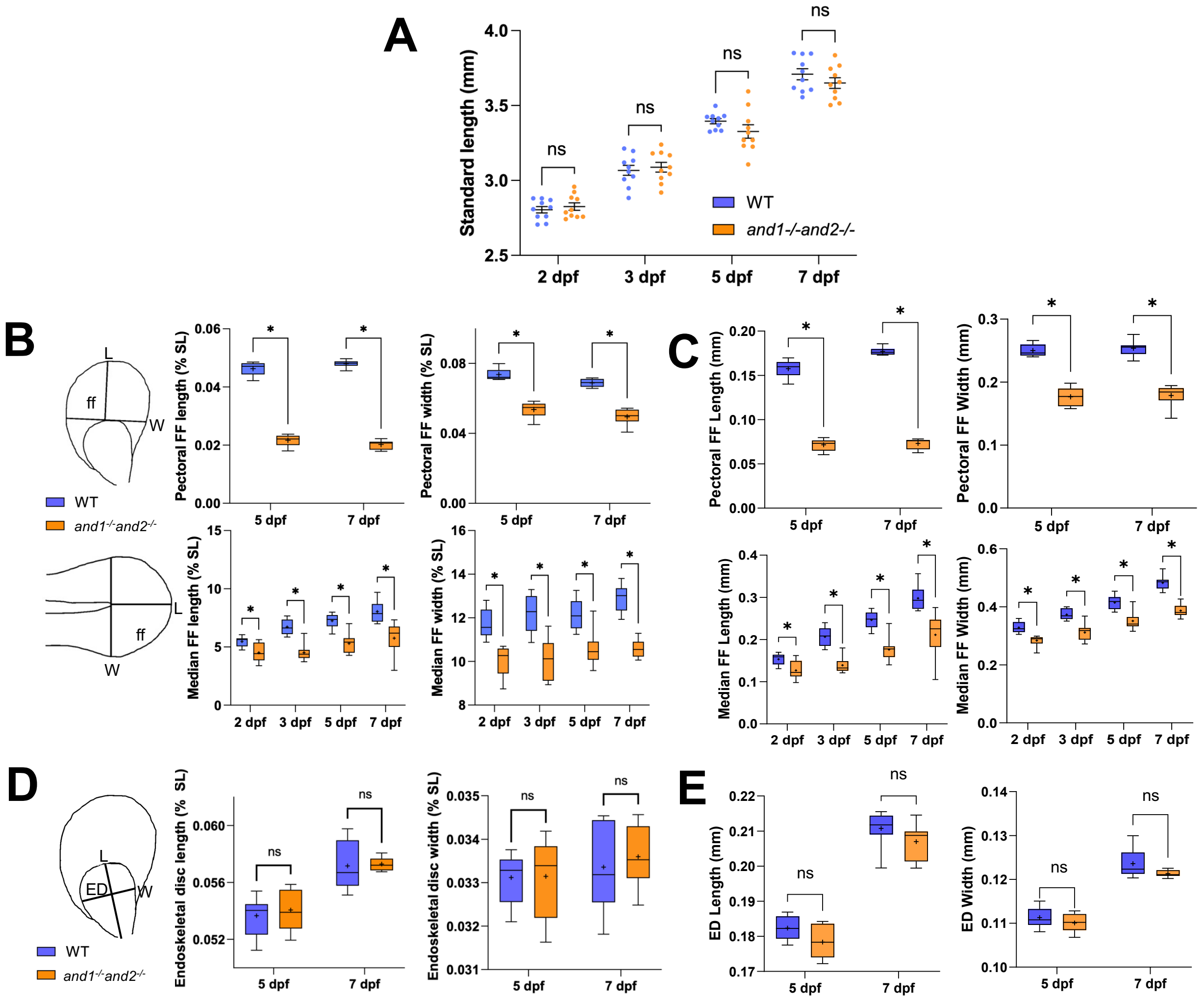

Fig. S4. Fin fold and adult fin morphometric data.

(A) Standard length over time during early development of WT and mutant embryos and larvae (n = 10 fish per genotype, per time point). Plot shows means (+) +/- SEM. Dots indicate individual values. Statistics: unpaired two-tailed Mann-Whitney *u*-test, α = 0.05, FDR: 1%, ns = not significant.

(B,C) Lengths (L) and widths (W) of pectoral (n_{WT} = 8; $n_{and1\text{-}/\text{-}and2\text{-}/\text{-}}$ = 8) and median fin folds (n_{WT} = 10; $n_{and1\text{-}/\text{-}and2\text{-}/\text{-}}$ = 10) of WT and double mutant embryos and larvae during development, normalized to SL (B) and non-normalized in mm (C). Accompanying schematics (left) visualize measurements taken. ff = fin fold. Plots show means (+), first and third quartiles (box), and lowest and highest values (whiskers). Statistics: unpaired two-tailed Mann-Whitney *u*-test, FDR: 1%, $p < 0.05$ (*).

Development • Supplementary information

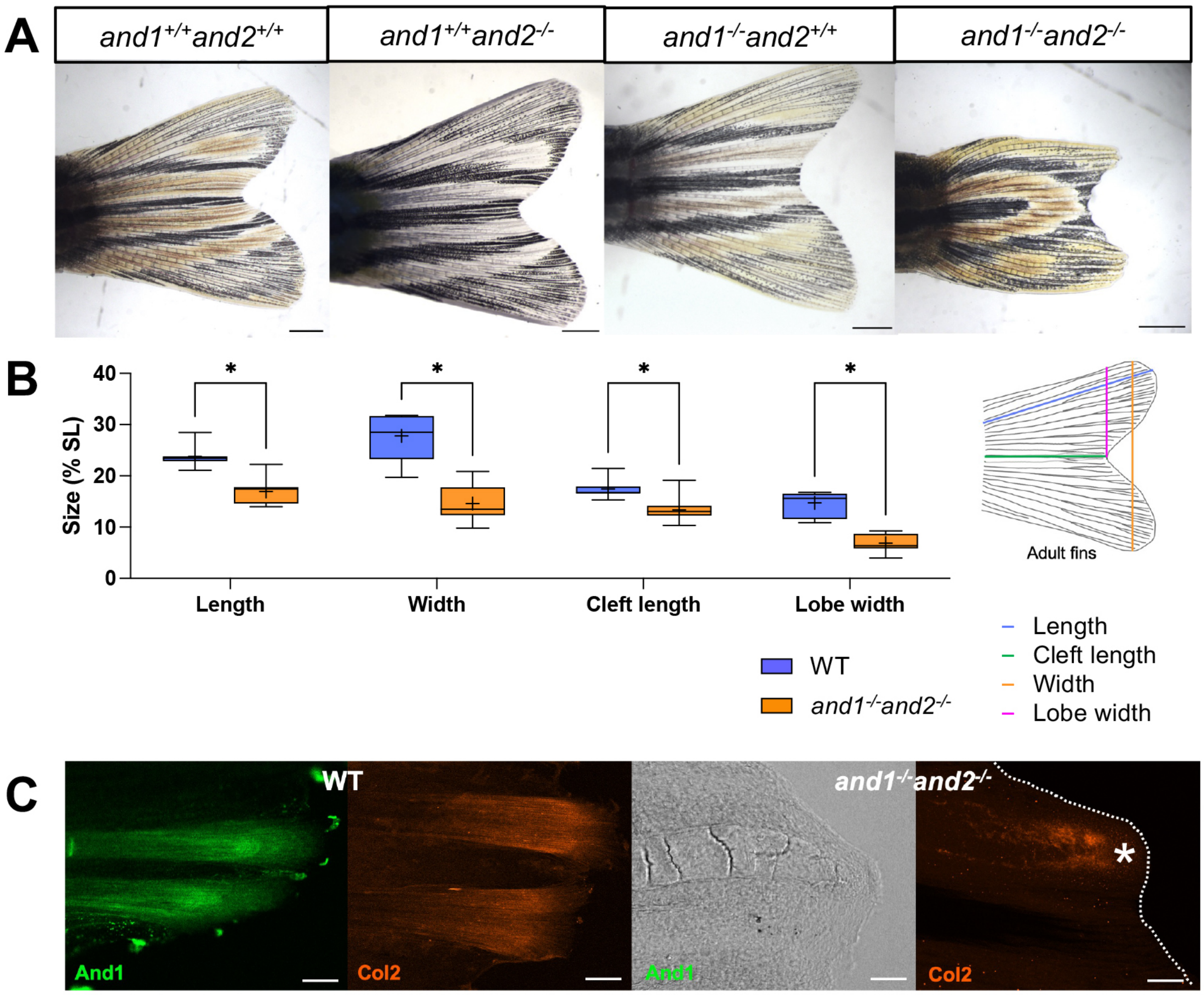

Fig. S5. Caudal fin geometry is affected in only adult double mutants. (A) Brightfield images of WT (n = 5 fish), *and1*$^{+/+}$*and2*$^{-/-}$ (n = 5 fish), *and1*$^{-/-}$*and2*+/+(n = 5 fish), and double mutant (n = 6 fish) caudal fins. Scale bars: 1 mm.

(B) Mean fin length, fin width, cleft length, and lobe width of WT (n = 7 fish) and double mutant (n = 12 fish) caudal fins at 140 dpf, normalized to SL. Accompanying schematic (right) shows measurements taken. Plots show means (+), first and third quartiles (box), and lowest and highest values (whiskers). Statistics: unpaired two-tailed Mann-Whitney *u*-test, $p < 0.05$ (*).

(C) Fluorescent channel maximum intensity projections of And1- and Col2-immunostained WT and double mutant adult caudal fin tips (also shown in Figure 4G-J). In WT rays, And1 (n = 5 fins) and Col2 (n = 5 fins) signal localizes to the actinotrichia (white dashed lines). In double mutants, no And1 signal is detected (a merged fluorescent-TD image is shown) (n = 5 fins), and Col2 is found at the tips of the blunt rays (n = 4 fins), not forming actinotrichia. White dotted outline represents margin of fin in the double mutant. Scale bars: 50 µm.

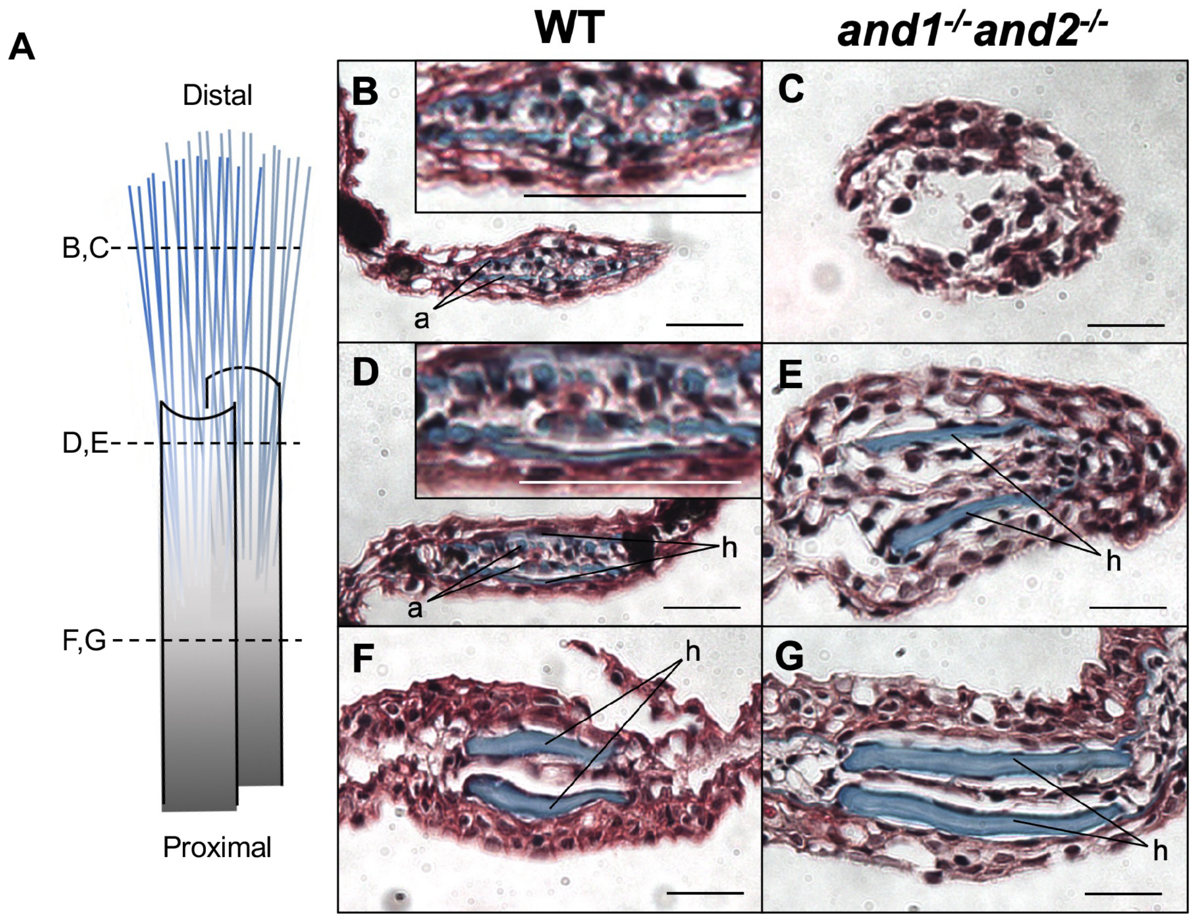

Fig. S6. Actinotrichia are not present at the distal tips in adult *and1$^{-/-}$and2$^{-/-}$* double mutant fin rays.

(A) Schematic of distal region of WT ray (also shown in Fig. 1B), depicting approximate positions (black dashed lines) of cross sections in (B-G).

(B-G) Masson's trichrome staining on 4 µm paraffin cross sections of the distal portion of intact adult WT sibling (B,D,F, n = 2 fins) and double mutant (C,E,G, n = 3 fins) anal fin rays. Collagenous material is stained blue, nuclei are stained dark red, and cytoplasm is stained a lighter red. Bundles of actinotrichia (a) are seen in two arrays in the WT (B). Inset in B shows a magnified view of the blue-stained actinotrichia bundles. There are no actinotrichia or collagenous material visible in the distalmost sections of double mutant ray epidermis (C). In the WT (D), the biconcave hemirays (h) enclose the two arrays of actinotrichia (magnified in inset). No actinotrichia are visible in the double mutant rays (E). More proximally, there are no actinotrichia in the WT (F) or the double mutant (G). The double mutants' wider bones are shown by the relatively wider hemirays, despite the proximal sections being at similar levels. Scale bars: 25 µm.

Development • Supplementary information

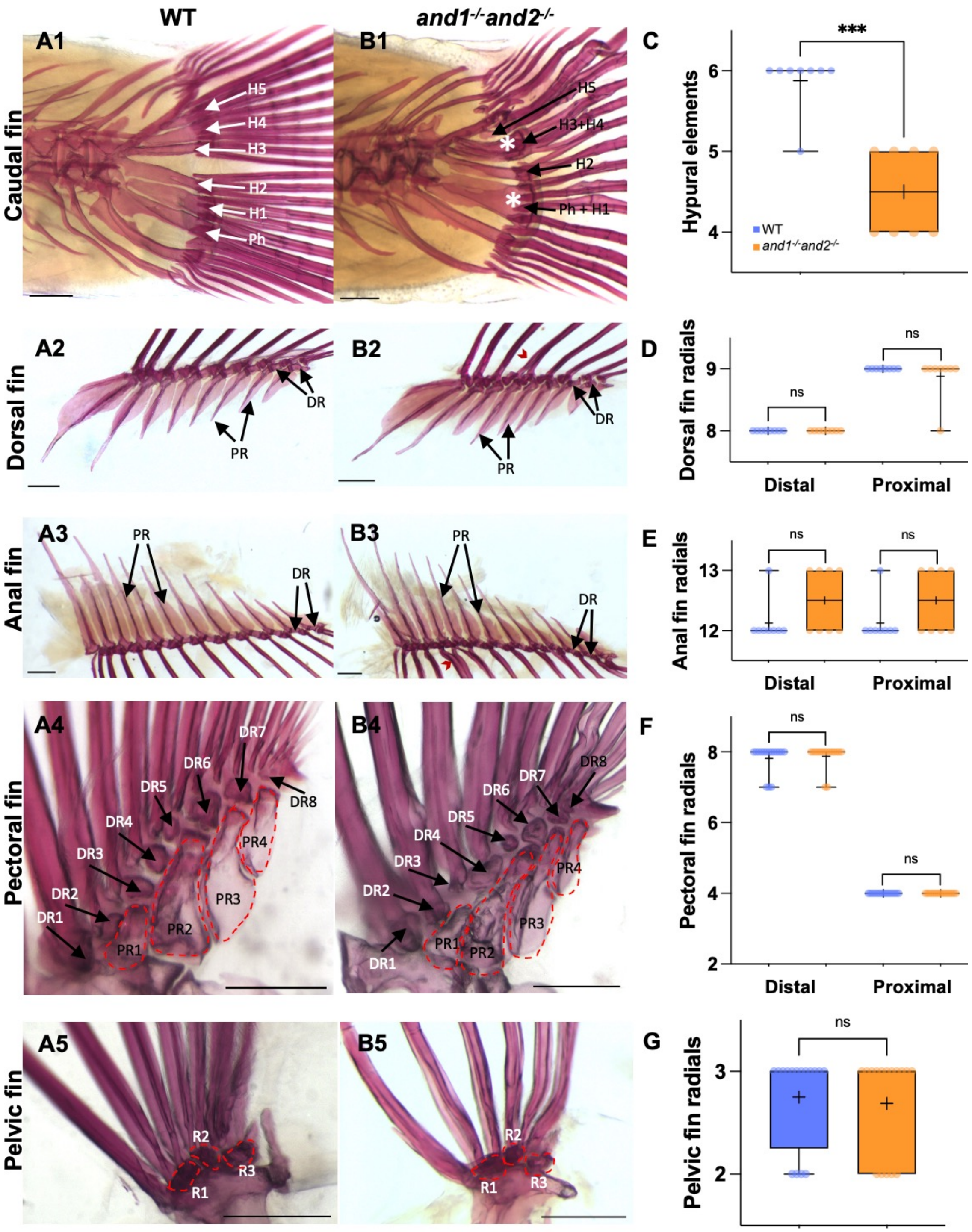

Fig. S7. Adult double mutants present with endoskeletal fusions in the caudal fin only. (A,B) Brightfield images of Alizarin red-stained adult WT sibling (A, n = 8) and double mutant (n = 8) (B) caudal (A1,B1), dorsal (A2,B2), anal (A3,B3), pectoral (A4,B4), and pelvic (A5,B5) fins.

Hypurals are fused in the caudal fin (white asterisks, B1). Fin rays occasionally fuse in the dorsal and anal fins (red arrowheads, B2,3). Red dotted outline emphasizes boundaries of radial elements (A4,5;B4,5). Images in panels B1 and B2 are also shown in Fig. 3B, with a focus on the rays. Ph: Parhypural; H: Hypural; DR: Distal radial; PR: Proximal radial; R: Pelvic radial. Scale bars: 500 µm.

(C-G) Mean (+) number of endoskeletal elements in WT sibling (n = 8) and double mutant (n = 8) caudal (C), dorsal (D), anal (E), pectoral (F), and pelvic (G) fins. Plots show means (+), first and third quartiles (box), and lowest and highest values (whiskers). Dots indicate individual values. Statistics: unpaired two-tailed Mann-Whitney *u*-test, $\alpha = 0.05$; $p = 0.0008$ (***), ns = not significant.

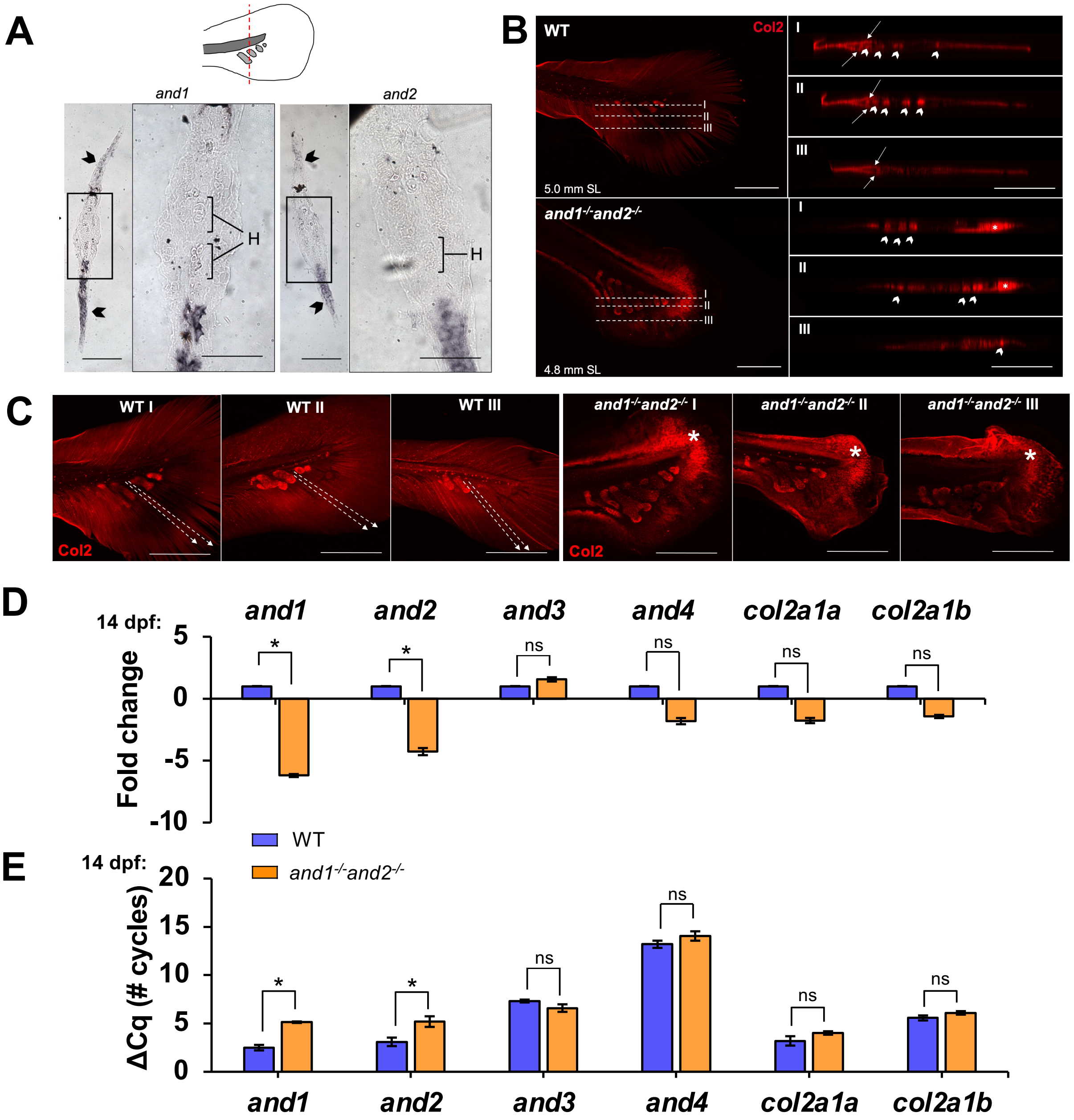

Fig. S8. Actinotrichia indirectly influence the developing caudal fin endoskeleton. (A) *In situ* hybridization of *and1* and *and2* riboprobes on 16 µm WT (4.5 mm SL) median FF transverse cryosections. Schematic shows approximate position of cross sections (red dashed line). Black arrowheads indicate expression in dorsal and ventral regions of fin folds. Area in black boxes is shown at a greater magnification. Brackets show cartilage hypural templates (H). Scale bars: 100 µm; magnified image scale bars: 50 µm.

Development • Supplementary information

(B) Dorsoventral Z-stack re-slices of anti-Col2 immunostaining on larval median FFs, through the developing hypural elements. Slices (right, **I-III**, dashed lines) show approximate position along scan. WT (n = 9 fish) shows the hypurals (white arrowheads) developing between two arrays of actinotrichia (white arrows), while the double mutants (n = 7 fish) show a disorganized arrangement of cartilage and Col2 aggregates (white asterisks), and Col2 does not contribute to two arrays of fibers. Scale bars: 200 μm.

(C) Maximum intensity projections of confocal Z-stacks of three representative Col2-immunostained WT sibling (n = 9 fish) and double mutant (n = 7 fish) posterior median FFs during cartilage development (between 4.5 mm SL and 5.2 mm SL). The hypural cartilages are seen among the actinotrichia (white dashed arrows) in WT larvae. Double mutants lack actinotrichia and have aggregation and disorganization of Col2 (white asterisks) within the FF, distal to the hypurals. Note: WT1 is also represented in Fig. 5**I**. Scale bars: 200 μm.

(D) Relative fold change of *and1*, *and2*, *and3*, *and4*, *col2a1a*, and *col2a1b* expression in WT sibling and double mutant tails at 14 dpf (n = 15 tails per biological replicate). WT expression level was set to 1. Fold change was determined by RT-qPCR using the ΔΔCq method. Error bars: Standard error of ΔΔCq. Statistics: unpaired two-tailed t-test, $p < 0.05$ (*).

(E) Mean ΔCq of *and1*, *and2*, *and3*, *and4*, *col2a1a*, and *col2a1b* expression in WT sibling and double mutant tails at 14 dpf (n = 15 tails per biological replicate). Error bars: Standard error of mean ΔCq. Statistics: unpaired two-tailed t-test, $p < 0.05$ (*).

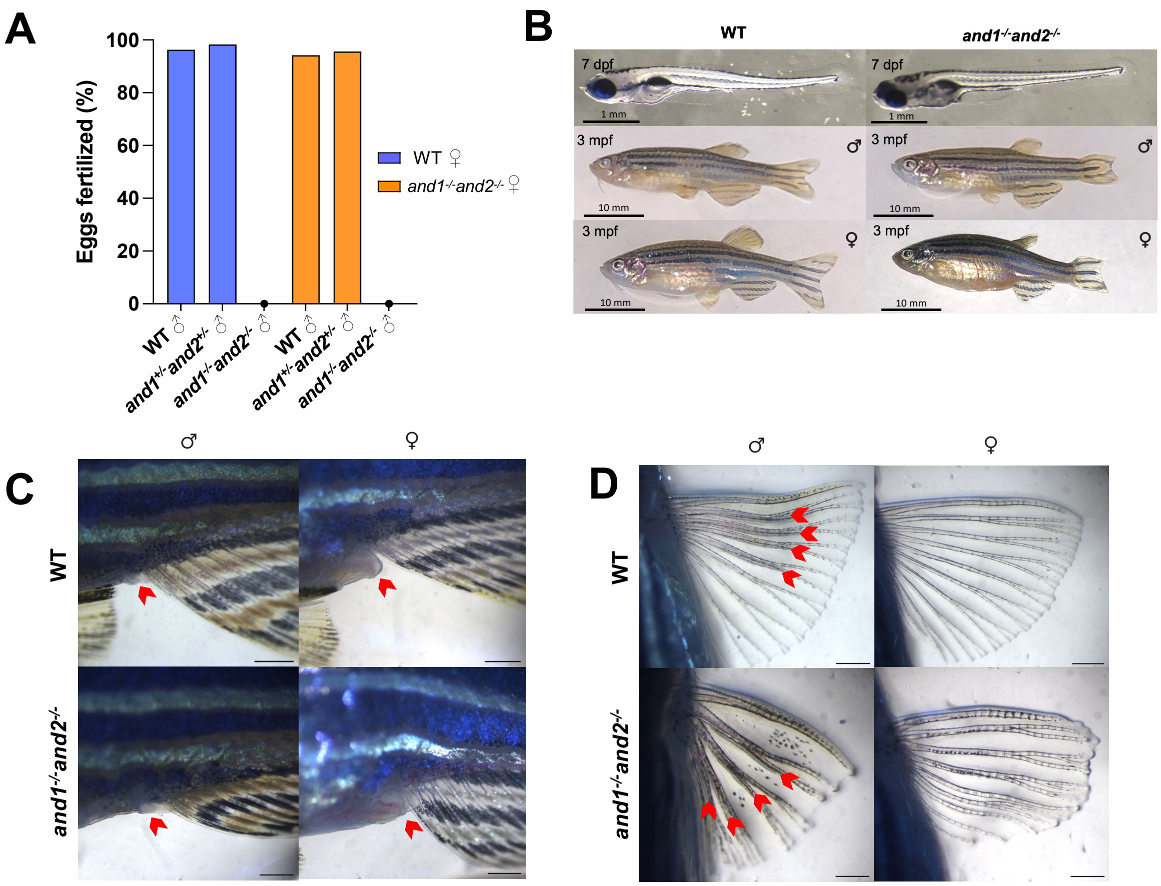

Fig. S9. Double mutants show no obvious defects outside the fins.

(A) Percentage of WT and double mutant eggs (n = 3 females) fertilized by WT, double heterozygous, and double homozygous males in breeding traps. Data pooled from 3 trials per cross. Males and females were arranged in 1:1 pairs (n = 3 couples) in breeding traps.

(B) Whole live WT and double mutant larvae at 7 dpf (top), and adult males (middle), and adult females (bottom) and 10 months post-fertilization (mpf).

(C) Brightfield images of WT and double mutant male and female urogenital pores (red arrowheads). Males do not have an obvious protrusion (left), whereas females (right) have urogenital papillae in both WT and double mutant fish.

(D) Brightfield images of WT and double mutant male and female pectoral fins. Pectoral breeding tubercle clusters (red arrowheads) are present in males (left) and absent in females (right) of both genotypes.

Development • Supplementary information

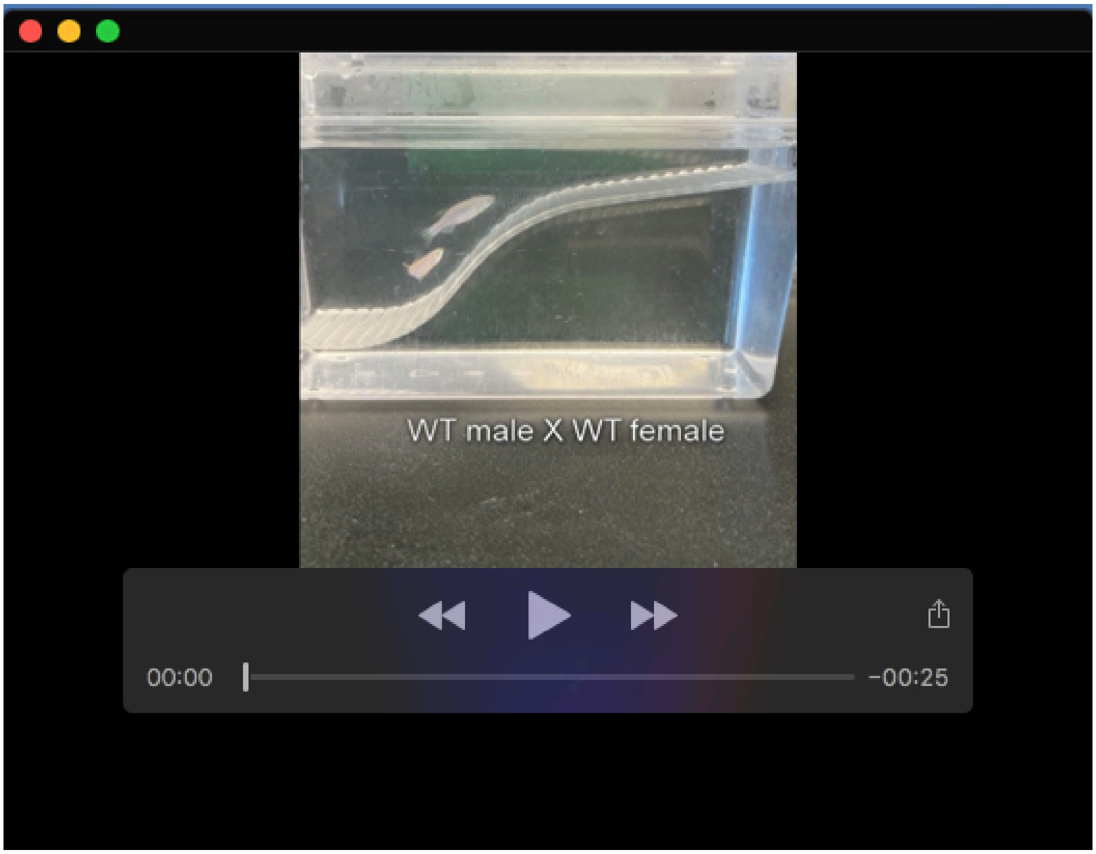

Movie 1. Courtship behaviour of WT and double mutant male zebrafish. WT and double mutant males were each crossed 1:1 with WT females (n = 3 pairs). One WT male X WT female sequence is shown, followed by two double mutant male X WT female sequences. In each sequence, a full-speed lateral view is shown first, then slowed to 25%, with annotations indicating the following behaviours: 2) Escorting, 3) Encircling, 4) Quivering, 5) Wrap-around, and 6) Spawning. Chasing is not presented for video brevity, as Chasing and Escorting can occur multiple times before the sequence continues. An overhead view of the Wrap-around behaviour follows, slowed to 10%. WT males participate in all phases of courtship behaviour, resulting in egg release. In the double mutant male courtship sequence 1, males participate in behaviours 2-4, with a clear Wrap-around attempt and bending of the female, but is unable to stimulate egg release. In Sequence 2, the male is shown attempting to Wrap-around the female many times without success, as he is not able to grasp her, due to his pectoral fin defects.

Development • Supplementary information
